# Differential effects of familial Alzheimer’s disease-causing mutations on amyloid precursor protein (APP) trafficking, proteolytic conversion, and synaptogenic activity

**DOI:** 10.1186/s40478-023-01577-y

**Published:** 2023-06-01

**Authors:** Sandra Schilling, Ajay Pradhan, Amelie Heesch, Andrea Helbig, Kaj Blennow, Christian Koch, Lea Bertgen, Edward H. Koo, Gunnar Brinkmalm, Henrik Zetterberg, Stefan Kins, Simone Eggert

**Affiliations:** 1 Department of Human Biology and Human Genetics, University of Kaiserslautern, 67663 Kaiserslautern, Germany; 2 Present Address: Department of Neurogenetics, Max Planck Institute for Multidisciplinary Sciences, City-Campus, Hermann-Rein-Str. 3, 37075 Göttingen, Germany; 3 Department of Psychiatry and Neurochemistry, Institute of Neuroscience and Physiology, The Sahlgrenska Academy at the University of Gothenburg, Mölndal, Sweden; 4 Clinical Neurochemistry Laboratory, Sahlgrenska University Hospital, Mölndal, Sweden; 5 Department of Neurodegenerative Disease, UCL Institute of Neurology, Queen Square, London, UK; 6 UK Dementia Research Institute at UCL, London, UK; 7 Hong Kong Center for Neurodegenerative Diseases, Clear Water Bay, Hong Kong, China; 8 San Diego (UCSD), Department of Neuroscience, University of California, La Jolla, CA 92093-0662 USA

**Keywords:** Alzheimer’s disease, Amyloid precursor protein, Familial Alzheimer disease, Beta Amyloid, Trafficking, Processing

## Abstract

The amyloid precursor protein (APP) is a key player in Alzheimer`s disease (AD) and the precursor of the Aβ peptide, which is generated by consecutive cleavages of β- and γ-secretases. Familial Alzheimer’s disease (FAD) describes a hereditary subgroup of AD that represents a low percentage of AD cases with an early onset of the disease. Different APP FAD mutations are thought to have qualitatively different effects on its proteolytic conversion. However, few studies have explored the pathogenic and putative physiological differences in more detail. Here, we compared different FAD mutations, located at the β- (Swedish), α- (Flemish, Arctic, Iowa) or γ-secretase (Iberian) cleavage sites. We examined heterologous expression of APP WT and FAD mutants in non-neuronal cells and their impact on presynaptic differentiation in contacting axons of co-cultured neurons. To decipher the underlying molecular mechanism, we tested the subcellular localization, the endocytosis rate and the proteolytic processing in detail by immunoprecipitation–mass spectrometry. Interestingly, we found that only the Iberian mutation showed altered synaptogenic function. Furthermore, the APP Iowa mutant shows significantly decreased α-secretase processing which is in line with our results that APP carrying the Iowa mutation was significantly increased in early endosomes. However, most interestingly, immunoprecipitation–mass spectrometry analysis revealed that the amino acid substitutions of APP FAD mutants have a decisive impact on their processing reflected in altered Aβ profiles. Importantly, N-terminally truncated Aβ peptides starting at position 5 were detected preferentially for APP Flemish, Arctic, and Iowa mutants containing amino acid substitutions around the α-secretase cleavage site. The strongest change in the ratio of Aβ40/Aβ42 was observed for the Iberian mutation while APP Swedish showed a substantial increase in Aβ1–17 peptides. Together, our data indicate that familial AD mutations located at the α-, β-, and γ-secretase cleavage sites show considerable differences in the underlying pathogenic mechanisms.

## Background

Alzheimer´s disease (AD) is the most prevalent form of dementia and involves the amyloid precursor protein (APP) playing a key role in the pathology of AD [1, 2]. APP, a type I transmembrane protein, is trafficked through the secretory pathway and is proteolytically converted in different cellular compartments [3]. APP can be processed by both, α- and β-secretase, whereby two large extracellular fragments (sAPPα and sAPPβ) with a size difference of 16 amino acids are secreted [4, 5]. The remaining C-terminal fragments (α-CTF and β-CTF) are subsequently cleaved by γ-secretase within the transmembrane domain [6]. This leads to the release of the APP intracellular domain (AICD) as well as to the secretion of the small peptides p3 (from α-CTF) or Aβ (from β-CTF) [7]. γ-Secretase starts cleaving APP at position 48/49 (Aβ numbering) [7, 8]. The sequential cleavages are carried out in two product lines. The major pathway is the Aβ40 line starting from Aβ49—Aβ46—Aβ43—Aβ40 to Aβ37 and the minor Aβ42 line starting from Aβ48—Aβ45—Aβ42 to Aβ38. For APP WT, Aβ42 represents 10% of the generated Aβ peptides while Aβ40 is produced to an extent of ~ 80% [9, 10]. Importantly, Aβ42 is more prone to form aggregates than Aβ40 due to two additional hydrophobic amino acids and therefore represents the main component of plaques in AD patients [11, 12].

AD mainly occurs in a sporadic form, but also has a genetic origin which is inherited in an autosomal dominant fashion, the so called familial form (familial Alzheimer`s disease, FAD) leading to an early onset of the disease [13].

Three genes were identified to be involved in the pathogenesis of FAD: *APP*, presenilin-1 and presenilin-2 (*PSEN1 and PSEN2*) [13]. PSEN1 and PSEN2 are both reported to be part of the γ-secretase complex [14, 15]. FAD amino acid substitutions in APP are located around α-, β- and γ-secretase cleavage sites. A double variant at the β-secretase cleavage site occurring in a Swedish family (KM670/671NL according to numbering of APP695 or position − 1/ − 2, Aβ numbering) has been reported to lead to a 4—sevenfold increased production of total Aβ [16–18]. FAD amino acid changes were also found close to the α-secretase cleavage site, like *APP* Flemish (A692G, A21G), *APP* Arctic (E693G, E22G) and *APP* Iowa (D694N, D23N) [18–20]. These amino acid substitutions are known to increase the aggregation properties of Aβ [21–24] and therefore also show toxicity to cerebrovascular cells, which leads to cerebral amyloid angiopathy (CAA) [19, 25, 26]. Further studies indicated a ~ twofold increase in Aβ40 and Aβ42 production for *APP* Flemish [18, 24, 27] and for *APP* Arctic significantly decreased Aβ42 levels while Aβ40 production was unchanged [18]. In contrast, for *APP* Iowa both, generation of Aβ40 and Aβ42 were not affected [24].

APP FAD amino acid substitutions, which are located around the γ-secretase cleavage site influence γ-secretase processing leading to an elevated Aβ42/Aβ40 ratio [13]. *APP* Iberian (I716F, I45F) has the strongest known impact by showing a 34-fold increased ratio of Aβ42/Aβ40 [28, 29].

So far, processing of the aforementioned *APP* FAD mutants has not been extensively studied [27, 30]. For *APP* Flemish, an increased Aβ/p3 ratio has been suggested without any quantitative analysis, which might indicate also decreased processing by α-secretase for this mutant [31]. However, a direct comparison of processing products of *APP* Swedish, Flemish, Arctic, Iowa, and Iberian has not been performed, yet.

Despite initial assumptions, mainly based on analyses on younger *APP* single KO mice, accumulating evidence suggests a pivotal role of APP at the synapse [32]. A part of this function directly depends on sAPPα generation [33, 34] while another part depends on APP full length synaptogenic activity, likely mediated via *trans*-synaptic signaling [35–37]. Although it seems obvious that FAD mutation in *APP* might also affect its physiological function, this has not been addressed, so far.

## Methods

### Plasmids

Generation of the myc FAD APP695 pcDNA3.1 + neo constructs was based on site directed mutagenesis and confirmed via sequencing.

### Immunocytochemistry

HeLa cells were seeded at a density of 35,000 cells per 24-well plate (Greiner) on 14-mm coverslips and transfected via jetPrime. The cells were fixed after 18–20 h for 10 min at 37 °C in 4% PFA with 4% sucrose and permeabilized for 10 min with 0.1% NP40. After incubation of primary antibodies (α-GM130, mouse, BD Bioscience and α-EEA1, mouse, BD Bioscience) at 4 °C overnight and secondary antibodies for 1 h at room temperature (RT) (Alexa Flour 488 and 594) cells were embedded in Mowiol (Sigma-Aldrich) and subjected to imaging with the software Axiovision 4.8 at the microscope Axio Observer Z.1.

### Analysis of proteolytic processing

HEK293T cells were transiently transfected at a confluency of 70% with jetPRIME (PolyPlus) according to manufacturer instructions with NT myc tagged APP and APP FAD mutants. The media were conditioned for 2 h (1 ml of media in one well of a 6-well plate). The cells were harvested and lysed in 200 µl lysis buffer [50 mM Tris/HCl, pH 7.5; 150 mM NaCl; 5 mM EDTA; 1% NP-40; 1:25 Complete Protease Inhibitor (with EDTA), Roche] for 20 min. Cell debris was pelleted at 15,700 × *g* for 10 min at 4 °C. For analysis of the CTFs, the same amounts of protein were loaded on a 4–12% Bis–Tris gel (Invitrogen). To investigate shedding of the FAD mutants, cell lysates as well as conditioned media were separated on 8% Tris/glycine gels and visualized via 22C11 (1:2000; mouse monoclonal), WO2 (1 µg/ml, mouse monoclonal), anti-sAPPβ (1:1000, rabbit polyclonal, IBL) or Y188 (1:5000, rabbit monoclonal, Abcam) followed by secondary antibodies, which are coupled to HRP for detection after Western blotting with ECL (Pierce). Images were analyzed via ImageJ, and the ratio between CTF or secreted proteins and full-length proteins was quantified. Statistical analysis was performed using ANOVA and Tukey’s post hoc test.

### Antibody uptake assay

N2a cells were cultured in MEM media with 10% (v/v) FBS, 1% (v/v) Pen/Strep, 1% (v/v) nonessential amino acids, 1% (v/v) sodium pyruvate, and 2 mM L-glutamine. A total of 70,000 cells were plated per 14 mm coverslip (Marienfeld) in a 24-well plate that had been coated with poly-L-lysine in double-distilled H_2_O. The cells were transfected the following day with jetPRIME (PolyPlus) according to manufacturer instructions with APP, APP FAD mutants and APP ΔCT, which served as a negative control. Seventeen to 24 h after transfection, the cells were placed on ice to stop endocytosis. After washing with OptiMEM (Invitrogen), the cells were incubated for 30 min with the primary α-c-myc antibody (1:200; 9E10, mouse monoclonal, Abcam) against the myc tag of the transfected proteins. The cells were washed with OptiMEM again to remove the unbound antibody. Subsequently, endocytosis was allowed at 37 °C for different time points in prewarmed N2a growth media. Afterwards, the cells were cooled down again to 4 °C and fixed with 4% (w/v) PFA with 4% (w/v) sucrose. After permeabilization for 10 min with 0.1% (v/v) NP-40 in 1 × PBS, the cells were blocked in 5% (v/v) goat serum in 1 × PBS and incubated with Alexa Fluor 594-conjugated secondary antibody (1:400; Invitrogen) to stain the remaining Proteins at the cell surface and the internalized protein. After a further washing step with 1 × PBS, the coverslips were embedded in Mowiol. The z-stack images were taken with the Axio Observer Z.1 Microscope (with apotome; Zeiss). Projections of these stacks were performed with the program ImageJ. For each construct and time point, the amount of endocytosed protein was determined by measuring the intensity of the internalized protein and the intensity of the surface protein. The ratio of the endocytosed protein to total intensities (internalized plus cell surface protein) represents the amount of endocytosed protein per cell. Statistical analysis was performed using one-way ANOVA followed by Tukey’s post hoc test or if not normally distributed by Kruskal–Wallis test followed by Dunn’s multiple-comparison test (*n* ≥ 4; *p < 0.05; **p < 0.01; ***p < 0.001; bars represent the mean ± SEM).

### Cell surface biotinylation

5 × 10^5^ HEK293T cells were seeded on a 6-well plate. The following day the cells were transiently transfected with JetPrime with APP and FAD mutants. After 17–24 h, the cells were rinsed twice with ice cold 1 × PBS and incubated for 30 min at 4 °C with 1 ml of EZ-Link Sulfo-NHS-LC-Biotin (2 mg/ml; Pierce) in ice-cold PBS to biotinylated surface proteins. To quench unconjugated biotin, the cells were washed three times with 1 × PBS supplemented with 100 mM glycine. Cells were lysed in 1 × RIPA buffer [20 mM Tris/HCl, pH 8.0; 150 mM NaCl; 1% NP-40 (w/v); 0.5% deoxycholate; 5 mM EDTA, pH 8.0; 0.1% SDS; 1:25 Complete Protease Inhibitor (with EDTA), Roche)], and 20 µg of lysate were used for the direct load. Equal protein amounts were incubated with NeutrAvidin Agarose Resin (Pierce) overnight at 4 °C. On the following day, the beads were washed with RIPA buffer and boiled at 95 °C for 5 min in 2 × sample buffer with DTT to recover the biotinylated proteins. Direct load and surface proteins were separated on an 8% Tris/glycine gel and detected with αc-myc antibody.

### Blue native gel analysis

Blue native gel electrophoresis was performed according to a protocol modified from [38, 39]. In brief, cells in one 10-cm cell culture dish were washed once and collected in phosphate-buffered saline at 4 °C. The cell pellets were resuspended in 1 ml of homogenization buffer (250 mM sucrose in 20 mM HEPES, pH 7.4, with protease inhibitor mix “Complete”, Roche Rotkreuz, Switzerland) and then sheared by passing through a 27 × gauge needle 10 times. The postnuclear supernatant was collected after a low-speed spin at 300 × *g* for 15 min at 4 °C. The membranes were pelleted after centrifugation at 100,000 × *g.* for 1 h at 4 °C and washed once with 200 μl of homogenization buffer. After repeating the ultracentrifugation step, the pellets containing the membranes were resuspended in 200 μl of homogenization buffer.

Fifty microgram of protein was solubilized with Blue Native sample buffer (1.5 M amino caproic acid, 0.05 M Bis–Tris, 10% *n*-dodedecyl-β-D-maltoside, and protease inhibitor at pH 7.5). The samples were incubated on ice for 30 min and then centrifuged for 10 min at 14,000 rpm at 4 °C in a microcentrifuge. Blue Native loading buffer (5.0% Serva Coomassie Brilliant Blue G250 and 1.0 M aminocaproic acid) was added to the supernatant. The samples were separated on 4–15% Tris–HCl gels (Criterion, Bio-Rad) overnight at 4 °C with Coomassie Blue containing cathode buffer (10 × cathode buffer, pH 7.0, 0.5 M Tricine, 0.15 M Bis–Tris, 0.2% Coomassie Blue) and anode buffer (pH 7.0, 0.5 M Bis–Tris). The gel was transferred to a polyvinylidene difluoride membrane. The following molecular weight standards were used: thyroglobulin (669 kDa), apoferritin (443 kDa), catalase (240 kDa), aldolase (158 kDa), and bovine serum albumin (66 kDa), all from Sigma.

### Coculture assay (hemisynapse assay)

The synaptogenic activity of the *APP* gene family members was analyzed using a coculture assay with HEK293T (HEK) cells and primary cortical neurons [35, 40]. HEK293T cells were cultured in DMEM with 10% FBS and 1% Pen/Strep. The primary cortical neurons were prepared on E14 from C57BL/6 J mice. The cortices were dissociated with a fire-polished Pasteur pipette after incubation for 15 min at 37 °C in 0.05% trypsin–EDTA and washing five times with ice-cold HBSS supplemented with 10 mM HEPES. The isolated neurons were resuspended in DB1 media [DMEM with 10% (v/v) FCS, 0.79% (w/v) D-glucose, and 2 mM L-glutamine], and 140,000 cells/ml (500 µl/coverslip) were seeded on 14-mm coverslips (Marienfeld) pretreated with poly-L-lysine in borate buffer (20 µg/ml). After 6 h of incubation, the media was replaced by NM media [Neurobasal media with 2% (v/v) B-27 supplement and 2 mM glutamate]. At DIV6 of the neuronal culture, HEK293T cells were transiently transfected with jetPRIME (Polyplus). GFP pcDNA3.1( +) was used as a negative control, and Nlg1 HA pcDNA3.1( +) was used as a positive control. The *APP* FAD mutants were also analyzed. Twenty-four hours after transfection, the HEK cells were seeded at DIV7 on the neuronal culture at a density of 400,000 cells/coverslip. After a coculture time of 24 h, the cells were fixed at DIV8 for 10 min at 37 °C in 4% (w/v) PFA with 4% (w/v) sucrose and washed three times with 1 × PBS. Subsequently, an immunocytochemical staining was performed. The cells were permeabilized with 0.1% NP-40 in 1 × PBS for 10 min and blocked in 5% goat serum in 1 × PBS. The overexpressed proteins were visualized with an antibody against their HA tag (1:300; rat monoclonal (3F10), Roche) or α-c-myc (1:200, rat monoclonal, Serotec) followed by an Alexa Fluor 488-conjugated secondary antibody (1:1000; Invitrogen). For detection of a presynaptic marker, an antibody against synaptophysin (1:200, mouse monoclonal antibody, Sigma-Aldrich; secondary antibody Alexa Fluor 594 conjugated) was used. Synaptophysin staining was the readout of the assay. The synaptophysin puncta at the HEK cells were taken as an indication for presynaptic differentiation in the contacting neuron. For quantification, the number of synaptophysin puncta per cell was counted and the area that was covered by these puncta was determined. To avoid false-positive puncta, the assay was costained with an antibody against MAP2 (1:300; rabbit polyclonal, Santa Cruz Biotechnology; secondary antibody Alexa Fluor 647 conjugated). Only cells that did not contact MAP2-stained dendrites were chosen for quantification to ensure that only so-called hemisynapses between an HEK293T cell and an axon were analyzed. The z-stack images were taken with the Axio Observer Z.1 Microscope (with apotome; Zeiss), and quantification was performed via ImageJ analysis. Statistical analysis was performed using one-way ANOVA followed by Tukey’s post hoc test or if not normally distributed by Kruskal–Wallis test followed by Dunn’s multiple-comparison test (*n* ≥ 4; *p < 0.05; **p < 0.01; ***p < 0.001).

### Immunoprecipitation

Immunoprecipitation (IP) of conditioned cell media from transiently transfected HEK293T cells was performed using the method described previously with minor modifications [41]. In brief, 4 µg of antibodies 6E10 and 4G8 (BioLegend) were conjugated separately with Dynabeads M-280 Sheep Anti-Mouse IgG (Invitrogen) through an incubation period of one hour at room temperature. Phosphate buffered saline (PBS) was used as negative control and pooled human CSF was used as positive control. Cell media samples and the control samples were incubated overnight at 4 °C with 50 µl of the antibody-conjugated beads in the presence of 0.2% (w/v) Triton X-100 (Sigma Aldrich). King Fisher magnetic particle processor (Thermo Scientific) was used for automated elution of Aβ peptide consisting of sequential washes in 0.2% Triton X-100, PBS and 50 mM ammonium bicarbonate. The final eluates were collected in 100 µl 0.5% (v/v) formic acid (Fluka), dried in a vacuum centrifuge and kept at − 80 °C.

### Mass spectrometry

Mass spectrometry analysis using matrix-assisted laser desorption/ionization time-of-flight (MALDI-TOF) on the samples was performed as described previously with minor modifications [41]. Briefly, the samples were reconstituted in 5 µl of 0.1% formic acid/20% acetonitrile (v/v/v) via one hour incubation on a shaker at room temperature. A layer of α-cyano-4-hydroxycinnamic acid (CHCA) matrix (Bruker Daltonics) was first applied onto a steel MALDI target plate (Bruker Daltonics) and allowed to dry. The reconstituted samples were then mixed separately in CHCA matrix and applied on top of the dried spots in a sandwich scheme [41]. In order to account for spot-to-spot variability, each sample was spotted onto two different locations on the MALDI target plate as technical replicates. The mass spectra were collected using a RapiFlex instrument (Bruker Daltonics) in reflector mode. The obtained spectra were averages of 3000 laser shots fired randomly at the sample, 1000 shots at a time. Prior to acquisition, the instrument was mass calibrated using the Peptide Calibration Standard II (Bruker Daltonics) consisting of nine standard peptides. After acquisition FlexAnalysis 3.4 (Bruker Daltonics) was used to perform baseline subtraction and internal calibration on the unprocessed spectra to enable automatic peak area extraction with higher accuracy. For internal calibration, theoretical m/z values of Aβ1-15, Aβ1-16, Aβ1-17, Aβ1-19, Aβ1-38, Aβ1-39, Aβ1-40, and Aβ1-42 corresponding to the APP mutation type were used. The *relative* MALDI-TOF signal of each Aβ proteoform was calculated by dividing the peak area of each Aβ proteoform peak by the sum of the peak areas of all Aβ peak areas of Aβ1-15, Aβ1-16, Aβ1-17, Aβ1-19, Aβ1-38, Aβ1-39, Aβ1-40, which were present in all the samples.

### Statistical analysis

Statistical analysis was conducted in Graphpad Prism 9 using one-way Anova followed by post hoc tests with either Tukey’s test (for comparison between all samples) or Dunnett’s test (for comparison with *APP* WT).

## Results

### APP Iberian mutation affects APP synaptogenic activity

**Fig. 1:**
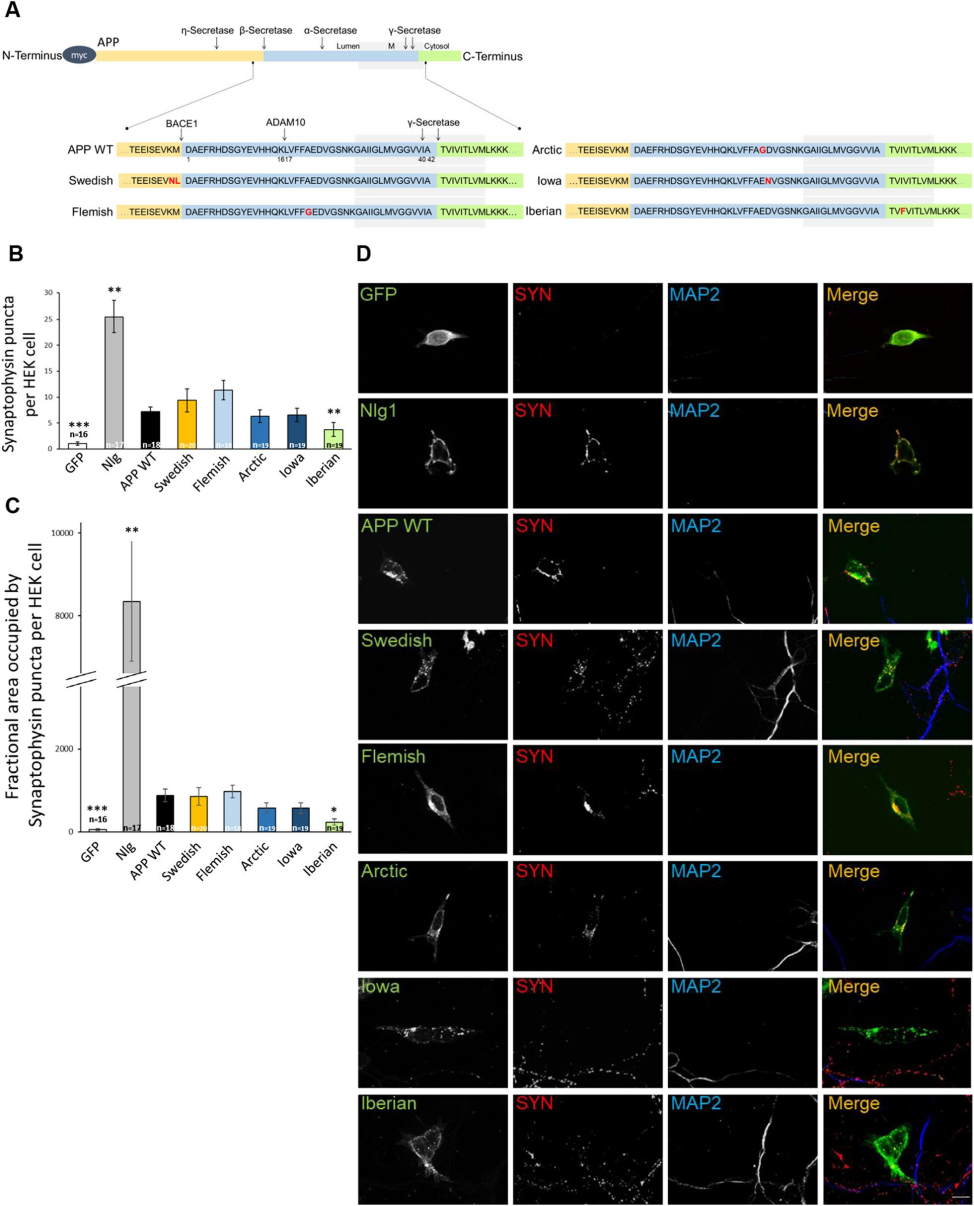
APP Iberian negatively impacts presynaptic differentiation. **A** The schematic representation shows APP WT and the APP FAD mutants which are analyzed. The FAD mutations were introduced into N-terminally c-myc-tagged APP695. The substituted amino acids are highlighted. The Aβ sequence is marked in blue and the transmembrane region in light grey (not to scale). Secretase cleavage sites are indicated by arrows. Numbers underneath the amino acids refer to their positions within the Aβ sequence. **B** HEK293T cells expressing c-myc-tagged APP WT, Swedish, Flemish, Arctic, Iowa, Iberian, neuroligin1 (positive control) or GFP (negative control) were seeded on WT primary cortical neurons (DIV7) and analyzed 24 h later via immunocytochemistry using anti-c-myc, anti-MAP2 (dendritic marker), and anti-synaptophysin (marker for presynaptic vesicles) antibodies. Scale bar, 10 µm. **C** Quantification of synaptophysin-positive puncta per HEK293T cell. **D** Quantification of synaptophysin-covered area per HEK293T cell. Bars represent mean values ± SEM of four independent experiments, *n* > 16/*N* = 4; Kruskal–Wallis test followed by Dunn’s multiple-comparison test; the * symbol represents significant differences in comparison with APP WT. *p < 0.05, **p < 0.01, and ***p < 0.005

We have shown previously that APP induces presynaptic differentiation in coculture assays [35, 36], presumably via *trans* interaction of APP with APP and APLP2 on the presynaptic side [42]. Therefore, we were curious to investigate the synaptogenic properties of APP containing FAD amino acid alterations compared with *APP* WT in this in vitro assay. We were examining one FAD amino acid substitution being closely located to the β-secretase cleavage site, APP Swedish [16] and one of the most prominent FAD amino acid changes at the γ-secretase cleavage site, the APP “Iberian mutation” [28, 29]. Furthermore, three APP FAD amino acid changes around the α-secretase cleavage site were analyzed APP “Flemish” [43], “Arctic” [44], and “Iowa mutation” [45]). All APP mutants were fused to an N-terminally located c-myc epitope (Fig. 1A) and were heterologously expressed in HEK cells. GFP- or neuroligin1 HA-expressing HEK cells served as negative and positive control, respectively.

The transfected HEK cells were seeded on primary cortical neuronal cultures at DIV7 and fixed and analyzed 24 h later (DIV8). The formation of presynaptic specializations was visualized via synaptophysin staining in axons, which contact the transfected HEK cells forming so-called hemi-synapses [35, 36, 40]. *APP* WT and *APP* FAD mutant expressing HEK cells were visualized using an α-c-myc antibody. Staining of the dendritic marker MAP2 was used to exclude the analysis of false-positive *bona fide* synapses between axons and dendrites (Fig. 1B). Firstly, synaptophysin positive *puncta* per HEK cell were quantified. Synaptic *puncta* represent clusters of synaptic proteins, in this case synaptophysin. The quantification revealed that the positive control neuroligin 1, a very well described synaptic adhesion molecule (SAM) [46], has a significantly higher induction rate of synaptic *puncta* than *APP* WT. Both, neuroligin1 as well as APP were showing significantly increased values of synaptophysin puncta compared with GFP transfected cells (Fig. 1B). Quantification of the area occupied by synaptophysin *puncta* revealed a significantly higher value for neuroligin 1 compared to *APP* WT while neuroligin 1 as well as APP had both significantly higher values compared with GFP transfected HEK cells (Fig. 1C). Interestingly, only APP carrying the Iberian mutation showed an impaired synaptogenic activity, as indicated by a significant reduction in synaptophysin *puncta* per cell as well as a reduced area of the synaptophysin staining. On the other hand, all other tested mutants, *APP* Swedish, Flemish, Arctic, and Iowa did not affect the synaptogenic activity of APP.

### The tested APP FAD mutants neither affect complex formation nor cell surface localization

**Fig. 2:**
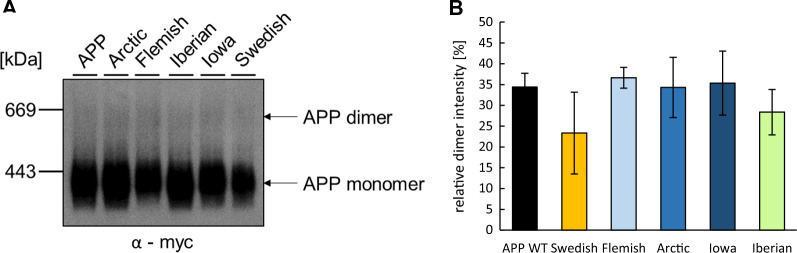
Analysis of dimer formation of APP WT and APP FAD mutants. **A** HEK293T cells were transiently transfected with N-terminally c-myc-tagged APP WT, Arctic, Flemish, Iberian, Iowa or Swedish. Cell lysates were prepared, and equal amounts of protein were analyzed via BN-PAGE and subsequent western blot detection. APP and the FAD mutants were detected with an anti-c-myc antibody. Full length APP and monomers/dimers are indicated with arrows. **B** BN-PAGE quantification. The relative dimer intensity represents the ratio of APP dimer intensity to the total APP signal intensity (APP dimer plus monomer). Bars represent mean values ± SEM; *n* = *3* (three biological replicates). Statistical analysis using one way ANOVA with Tukey`s post hoc test did not reveal significant differences

**Fig. 3:**
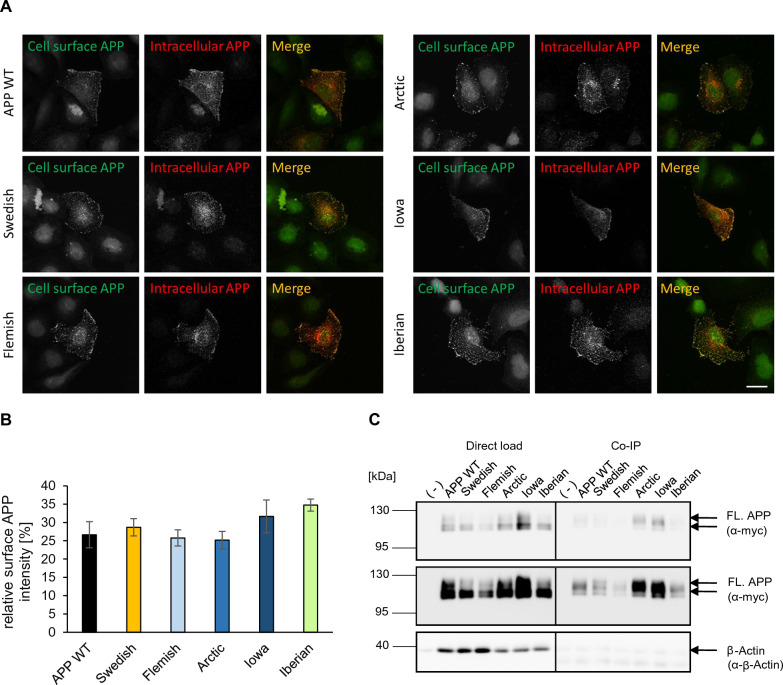
Presence of APP WT and APP FAD mutants at the cell surface. **A** Cell surface staining. HeLa cells were transiently transfected with N-terminally c-myc-tagged APP WT, Swedish, Arctic, Flemish, Iowa or Iberian. Cells were labeled with α-c-myc antibody (green, surface APP; red, total APP). The images were taken with an epifluorescence microscope including apotome. Scale bar: 20 μm. **B** ICC quantification. The relative surface APP intensity [%] indicates the percentage of APP present at the surface compared to total APP intensity. Bars represent mean values ± SEM; *n* = 18 (three biological replicates). Statistical analysis using one way ANOVA with Tukey`s post hoc test did not reveal any significant differences.** C** Cell surface biotinylation of transiently APP WT, Swedish, Arctic, Flemish, Iowa or Iberian transfected HEK293T cells. Direct load of cell lysates is shown in the left panel. In the right panel, APP cell surface levels after streptavidin immunoprecipitation and Western blot detection with anti-c-myc antibody are shown. β-Actin served as a loading control and was detected in the IP samples only to a minor extent, which confirms specificity of the pull down of surface proteins. The lower panel shows a higher exposure time

To test, if altered APP synaptogenic activity might be due to distinct complex formation all *APP* FAD mutants, were again heterologously expressed in HEK cells, and cell extracts were subjected to Blue Native gel analysis under semi-denaturing conditions. This method cannot distinguish between *cis* and *trans* dimers of APP or complexes with other APP interacting molecules. Equal protein amounts of cell lysates were examined, and APP complexes were solubilized using the mild detergent β-dodecyl maltoside. *APP* WT and the *APP* FAD mutants were detected using an α-c-myc antibody. Quantification revealed no significant changes between the *APP* WT and FAD mutants in high molecular complex formation (Fig. 2A, B). Furthermore, we were investigating the presence of *APP* WT and the *APP* FAD mutants immunocytochemically at the cell surface (Fig. 3A, B) as well as via cell surface biotinylation (Fig. 3C). No significant alterations regarding cell surface localization were observed for any of the *APP* FAD mutants compared to *APP* WT.

### None of the tested APP FAD mutants affect APP endocytosis

As differences in synaptogenic activity could be explained by distinct subcellular localization, we performed immunocytochemical analysis of HeLa cells heterologously expressing *APP* WT and the different *APP* FAD mutants. We performed the following immunocytochemical analysis also in HEK cells and obtained qualitatively the same result as in HeLa cells which were chosen for the quantitative analysis due to their larger cellular area.

**Fig. 4:**
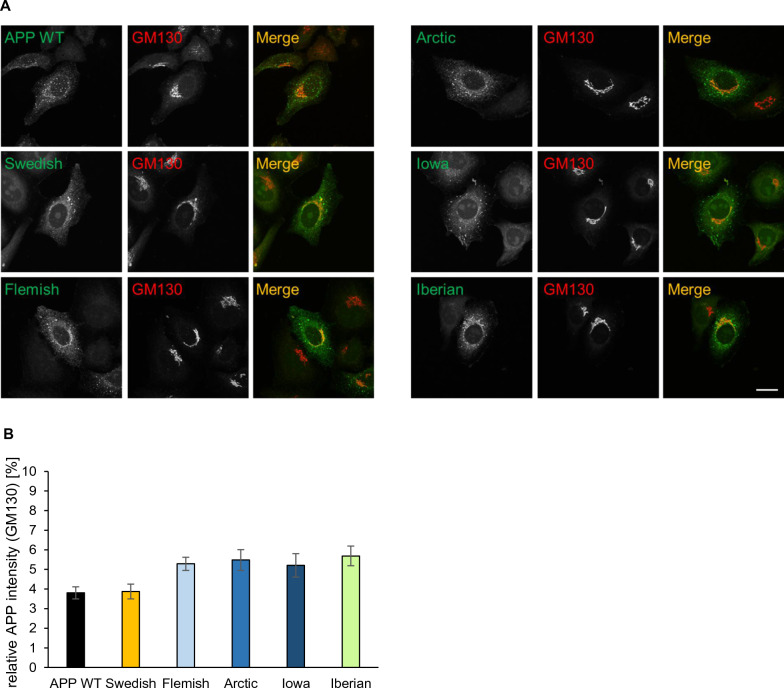
Colocalization of APP WT and APP FAD mutants in the *cis* Golgi apparatus. **A** HeLa cells were transiently transfected with N-terminally c-myc-tagged APP WT, Swedish, Arctic, Flemish, Iowa or Iberian. Cells were labeled with α-c-myc and α-GM130 (marker for Golgi apparatus) antibodies. The merge reveals colocalization of both channels, indicated in yellow. Scale bar: 20 μm. **B** ICC quantification. The relative mean integrated density [%] indicates the percentage of APP present in the *cis* Golgi apparatus. Bars represent mean values ± SEM; *n* = 30 (three biological replicates). Statistical analysis using one way ANOVA with Tukey`s post hoc test did not reveal any significant differences

**Fig. 5:**
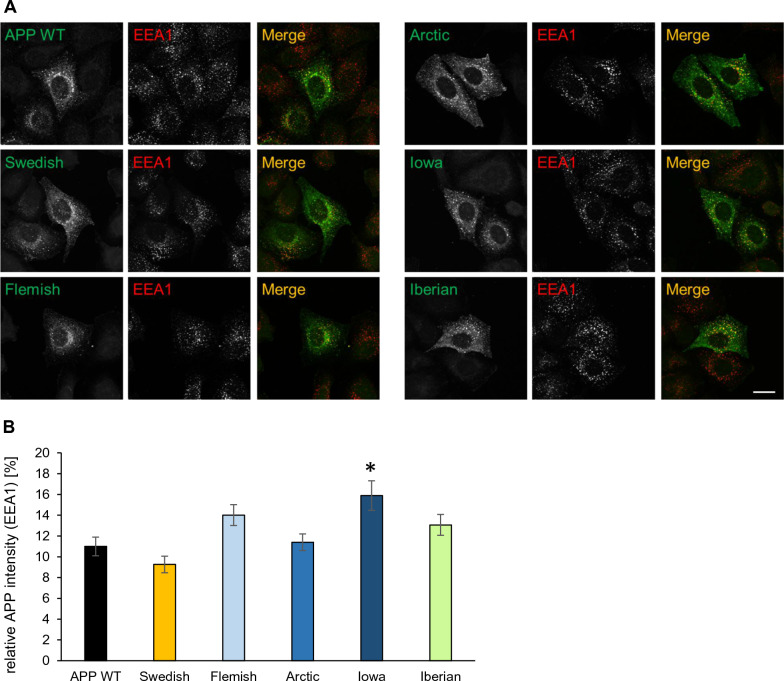
Colocalization of APP WT and APP FAD mutants in Early Endosomes. **A** HeLa cells were transiently transfected with N-terminally c-myc-tagged APP WT, Swedish, Arctic, Flemish, Iowa or Iberian. Cells were labeled with α-c-myc and α-EEA1 (marker for early endosomes) antibodies. The merge reveals colocalization of both channels, indicated in yellow. Scale bar: 20 μm. **B** ICC quantification. The relative mean integrated density indicates the percentage of APP present in Early Endosomes. Bars represent mean values ± SEM; *n* = 30 (three biological replicates). Statistical analysis was performed using one way ANOVA with Tukey`s post hoc test (*p < 0.05, **p < 0.01, ***p < 0.001)

**Fig. 6:**
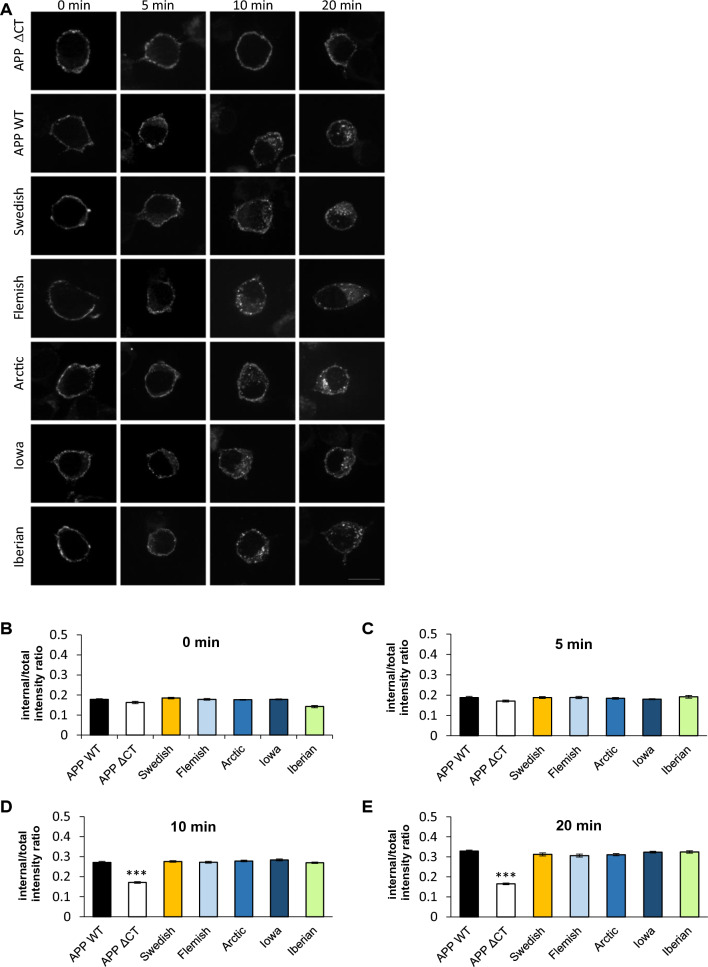
Internalization rate of APP WT compared to APP FAD mutants. An antibody-uptake assay was performed using APPΔCT as a negative control. **A** N2a-cells expressing N-terminally c-myc-tagged APP WT, Swedish, Flemish, Arctic, Iowa, Iberian or APPΔCT were incubated with a mouse anti-c-myc antibody at 4 °C. Subsequently, the cells were placed at 37 °C, allowing antibody uptake. After 0, 5, 10, or 20 min, cells were fixed and incubated with an anti-mouse Alexa Fluor 488-conjugated secondary antibody to mark surface APP. Afterwards, the cells were permeabilized and stained with an anti-mouse Alexa Fluor 594-conjugated secondary antibody. Representative immunofluorescence images of N2a cells transiently transfected with N-terminally c-myc APP WT or the indicated APP mutants showing only the signal of Alexa Fluor 594 representing endocytosed and remaining surface APP. Scale bar, 10 µm. **B** Quantification of internal/total intensity ratio for APP WT and the APP mutants analyzed at 0, 5, 10, and 20 min. Bars represent mean values ± SEM; *n* > 30 (three biological replicates). Statistical analysis was performed using Kruskal–Wallis test followed by Dunn’s multiple-comparison test; (*p < 0.05, **p < 0.01, ***p < 0.001)

The *APP* FAD mutants were visualized with an α-c-myc antibody (Figs. 4 and 5) and Golgi apparatus and early endosomes were stained with α-GM130 and α-EEA1 antibodies, respectively. Quantification of the colocalization of APP and the different *APP* FAD mutants with the *cis*-Golgi apparatus via Pearson coefficient revealed no significant changes (Fig. 4B), but interestingly, *APP* Iowa showed a significantly (p value = 0.03) stronger presence in early endosomes compared with *APP* WT (Fig. 5B). Since *APP* Iowa showed a significantly higher presence in endosomes, we analyzed the endocytosis rate of *APP* WT and the *APP* FAD mutants using an established antibody uptake assay [35, 47, 48] (Fig. 6A, B). N2a cells were chosen for the endocytosis assay since quantification of this assay is most reliable in this cell type due to the round cell shape. The time points 0, 5, 10, and 20 min were examined. All APP variants were heterologously expressed in N2a cells. The following day, the cells were incubated with an anti-c-myc (9E10) antibody at 4 °C to label surface APP. Afterwards, the cells were placed at 37 °C for the above-mentioned defined time points to allow endocytosis followed by fixation of the cells. Residual surface APP was visualized with a fluorescent secondary antibody Alexa Fluor 488. After permeabilization of the cells, endocytosed as well as remaining surface APP were labeled with a different secondary antibody, Alexa Fluor 594. This labeling allowed differentiation between surface APP and internalized APP. Here, only the signal of Alexa Fluor 594 is shown representing endocytosed APP and remaining cell surface APP. For quantification, the endocytosis rate (Endo) was determined by calculating the ratio of signal intensity of endocytosed APP (immunoreactivity of Alexa Fluor 594 for internal cell) to total intensity of the cell (immunoreactivity of Alexa Fluor 594 for the whole cell including the plasma membrane). After 20 min, APP WT was almost completely internalized (positive control). APPΔCT lacking the whole APP C-terminus including all known endocytosis motifs was used as a negative control [47]. No significant changes in their internalization rate were observed for the *APP* FAD mutants compared with *APP* WT (Fig. 6A, B).

These data show that the tested FAD mutations do not affect APP endocytosis rate. Thus, the Iowa mutation, causing altered localization of APP to endosomes, must be explained by different intracellular trafficking changes.

### The impact of ***APP*** FAD mutations on α- and β-secretase cleavage

**Fig. 7:**
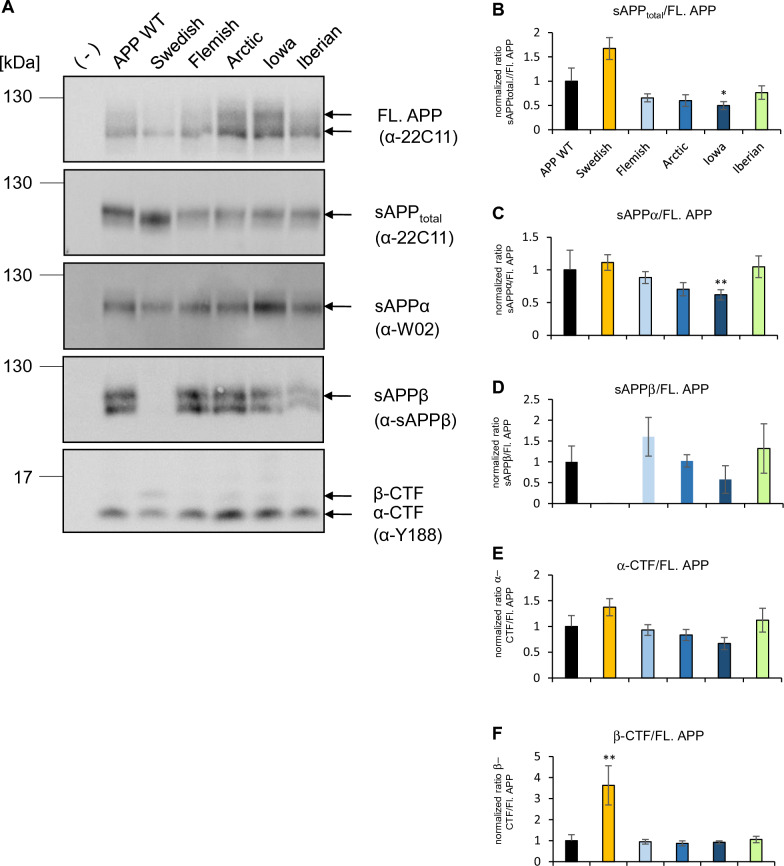
Analysis of the proteolytic processing of APP WT and APP FAD mutants. **A** HEK293T cells were transiently transfected with c-myc-tagged APP, Swedish, Flemish, Arctic, Iowa or Iberian. Equal volumes of cell lysates and conditioned media were analyzed via Western Blot. To detect APP full length protein, the cell lysate was analyzed with antibody 22C11. For detection of α-CTF and β-CTF, the lysate was examined with antibody Y188. The total amount of sAPP was detected with antibody 22C11 using conditioned media. The media was furthermore analyzed with antibodies W02 (to visualize sAPPα) and anti-sAPPβ. **B–F** Quantification of Western Blot signals from A. Normalized ratios from processed fragments to full length APP are shown (normalized to APP WT). Bars represent mean values ± SEM; *n* > 5 (5 biological replicates). Statistical analysis was performed using ANOVA test (*p < 0.05, **p < 0.01, ***p < 0.001)

Next, we analyzed the impact of the amino acid substitutions of the *APP* FAD mutants on their proteolytic conversion by α-, and β-secretase in comparison to *APP* WT. We performed western blot analyses to examine direct loads of cell lysates and media of heterologously expressing human HEK cells. Non-transfected HEK cells served as a control. Expression level of full length APP was visualized with an α-22C11 antibody and C-terminal fragments with an a-Y188 antibody. Media of the transfected HEK cells were investigated with the α-22C11 antibody to detect sAPP_total_, α-W02 antibody to visualize sAPPα, and an α-sAPPβ antibody to detect sAPPβ (Fig. 7A). For APP Swedish, carrying the amino acid substitution directly N-terminal to the β-secretase cleavage site, a significantly increased production of β-C-terminal fragments compared with *APP* WT was determined, as expected. Of note, sAPPβ fragments cannot be detected for *APP* Swedish with the sAPPβ antibody used since the two most C-terminal amino acids are altered. Furthermore, the secreted fragment of APP Swedish visualized with the antibody α-22C11 shows a small shift towards a lower apparent molecular weight compared to APP WT or the other APP proteins containing FAD amino acid substitutions (Fig. 7A). This may reflect the increased level of sAPPβ (which has a lower molecular weight than sAPPα) secreted from APP Swedish. Alternatively, the KM 670/671NL mutation in APP Swedish might also affect the apparent molecular weight of sAPPβ.

Interestingly, *APP* Iowa showed decreased sAPPα production, which is in line with the result of a higher colocalization of APP Iowa in endosomes (Fig. 7B). The other *APP* FAD mutants were not significantly altered compared with *APP* WT regarding α- or β-secretase cleavage of APP (Fig. 7 C-F).

### The impact of *APP* FAD mutations on Aβ generation

**Fig. 8:**
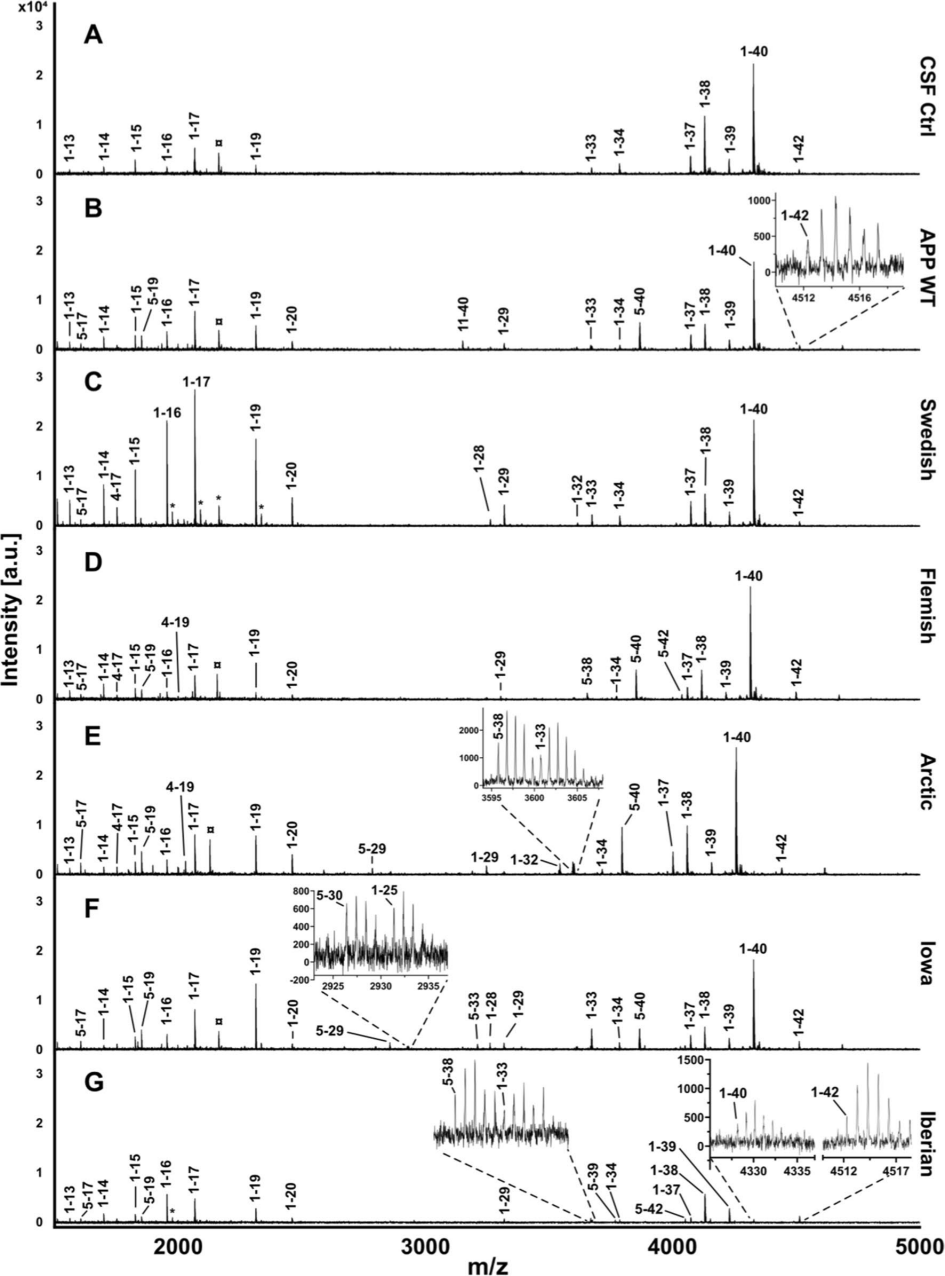
Analysis of Aβ proteoforms in *APP* WT and *APP* FAD mutants by immunoprecipitation-mass spectrometry. IP-MALDI-TOF on **A** human CSF positive control, **B** conditioned media from wild type HEK293T cells and transiently transfected HEK293T cells with** C** Swedish, **D** Iberian, **E** Iowa, **F** Flemish and **G** Arctic mutations yielded mass spectra of Aβ proteoforms. Data from only one biological replicate (out of two) are shown. ¤ indicates [Aβ1-40 + 2H]^2+^ and * represent peaks with unknown identities. Peak identity is considered confirmed when the m/z corresponds to the theoretical m/z of an Aβ proteoforms with a tolerance of 20 ppm. In the inserts, the arrows point to the monoisotopic peak of some interesting proteoforms

**Fig. 9:**
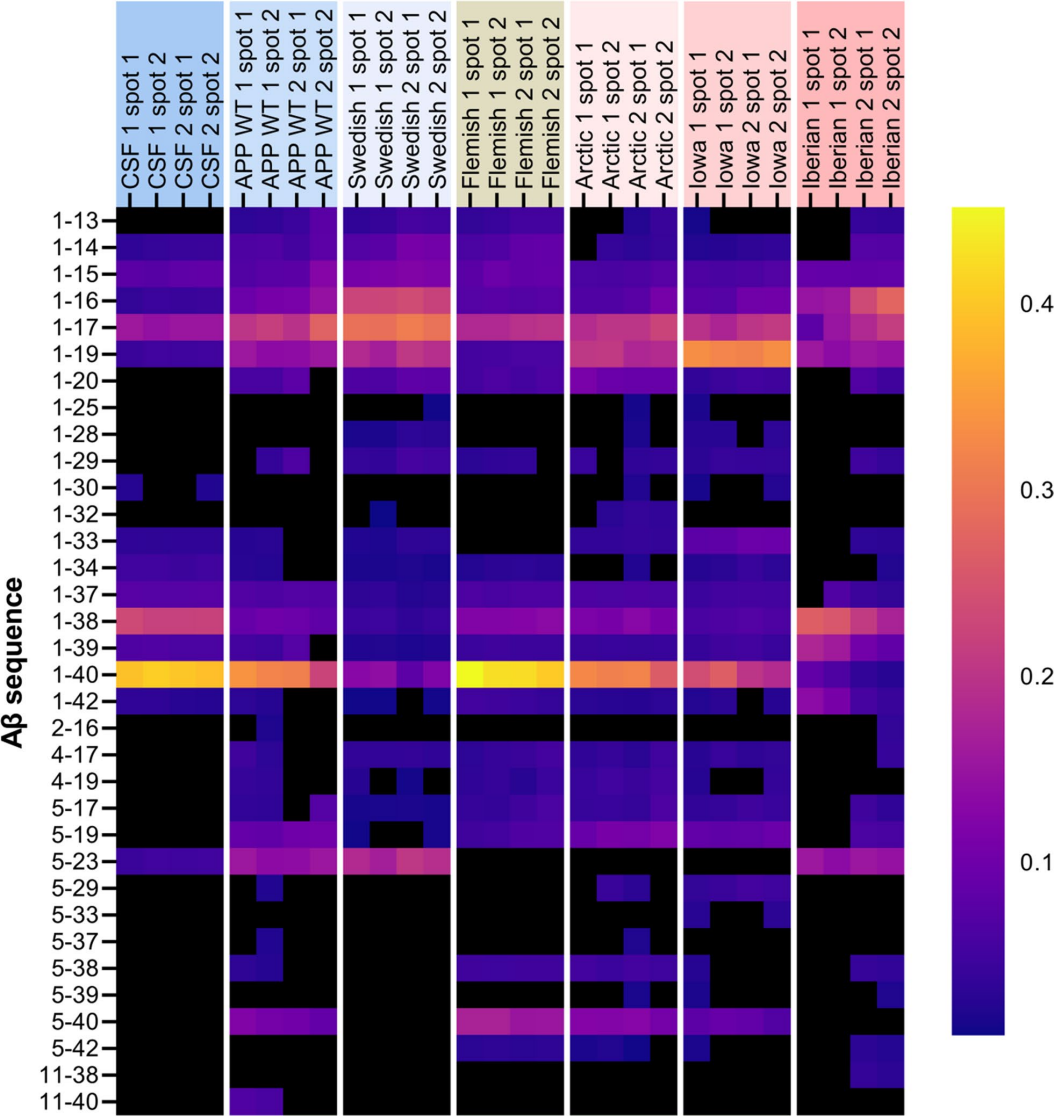
Normalized heatmap of Aβ peptide sequences observed during IP-MALDI-TOF on APP WT and APP FAD mutants. The four columns per sample type represent the two biological replicates (1 and 2) each with two technical MALDI-TOF replicates (spot 1 and spot 2). Normalization was performed by dividing peak area of individual Aβ proteoforms by the total peak area of all identified Aβ peaks in the sample’s spectrum. Black indicates the total absence of the peptide sequence in the spectrum, which may not necessarily always indicate the absence in the sample

**Fig. 10:**
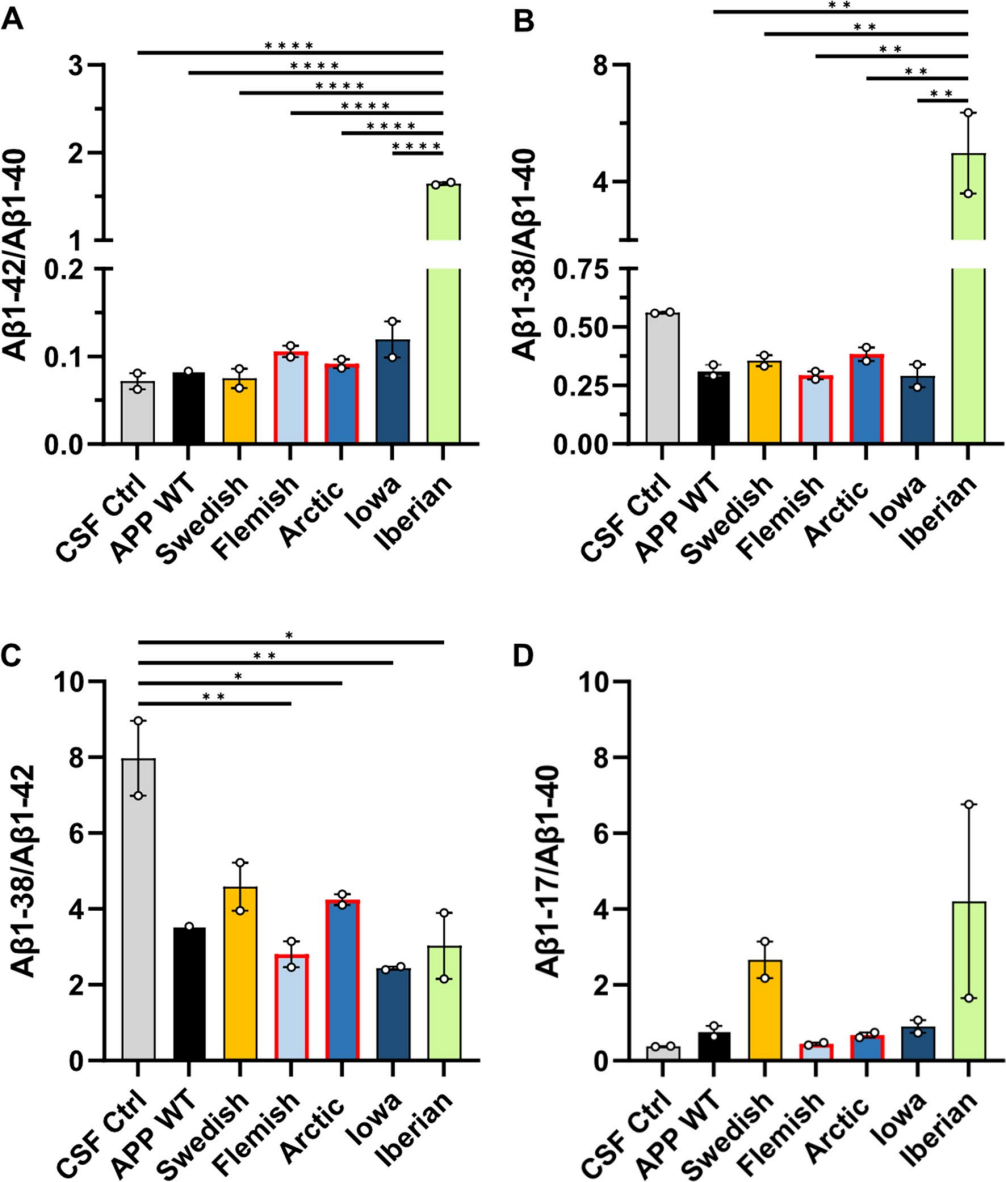
Comparison of Aβ proteoform ratios in cell media from *APP* WT and *APP* FAD mutants. The ratios of **A** Aβ1-42/Aβ1-40, **B** Aβ1-38/Aβ1-40 **(C)** Aβ1-38/Aβ1-42 and **(D)** Aβ1-17/Aβ1-40 were compared between *APP* WT and the FAD mutant HEK293T cells. The circles represent the two biological replicates (*N* = 2), each of which are the averaged technical replicates from MALDI-TOF. For comparing sample types, a one-way ANOVA with Tukey’s post hoc test was conducted (*p < 0.05, **p < 0.01, ****p < 0.0001)

Finally, we analyzed the impact of the amino acid substitutions of the APP FAD mutants on their proteolytic conversion by γ-secretase in comparison to APP WT. For this purpose, we analyzed the Aβ mass spectrometry profiles of *APP* FAD mutants for possible differences in the Aβ proteoform patterns. We investigated exactly the same media batches of heterologously expressing HEK cells as before for the western blot detection to perform IP-MALDI-TOF analyses. Antibodies 6E10 (epitope in the N-terminal region of Aβ aa Aβ4–8) and 4G8 (epitope C-terminal of the α-secretase cleavage site, aa Aβ18–22) were applied in combination for immunoprecipitation to capture Aβ proteoform fragments with both N- and C- terminal truncations. However, it is crucial to note that the amino acid substitution in Flemish (A21G) and Arctic (E22G) mutations reduces 4G8 antibody’s ability to properly bind to the epitope as reported by others [49–51] making the direct comparison of MALDI spectra with the other mutations more difficult. The representative MALDI mass spectra for the Aβ proteoforms secreted by the transfected HEK293T cells are shown in Fig. 8 and the heatmap of the normalized Aβ proteoforms is shown in Fig. 9. The important ratios of the peptides Aβ1-42/Aβ1-40, Aβ1-38/Aβ1-40, Aβ1-38/Aβ1-42 and Aβ1-17/Aβ1-40 were calculated to assist with comparison between the sample types (Fig. 10). The spectra of the CSF positive control containing human *APP* WT showed, among others, the familiar peaks of Aβ1-17, Aβ1-38, Aβ1-39, Aβ1-40, Aβ1-42 with Aβ1-40 being the major peak and Aβ1-42 one of the lowest (Figs. 8, 9). In comparison, the *APP* WT sample of the transfected HEK cells showed several prominent signals between Aβ1-17 and Aβ1-38, such as Aβ1-19, Aβ1-20, Aβ11-40, Aβ1-29, Aβ5-40 (Figs. 8, 9), and a similar Aβ1-42/Aβ1-40 ratio but lower Aβ1-38/Aβ1-40 and Aβ1-38/Aβ1-42 ratios (Fig. 10). The similarity in the Aβ secretion patterns suggests a successful transfection of the *APP* WT into the HEK cells.

**Fig. 11:**
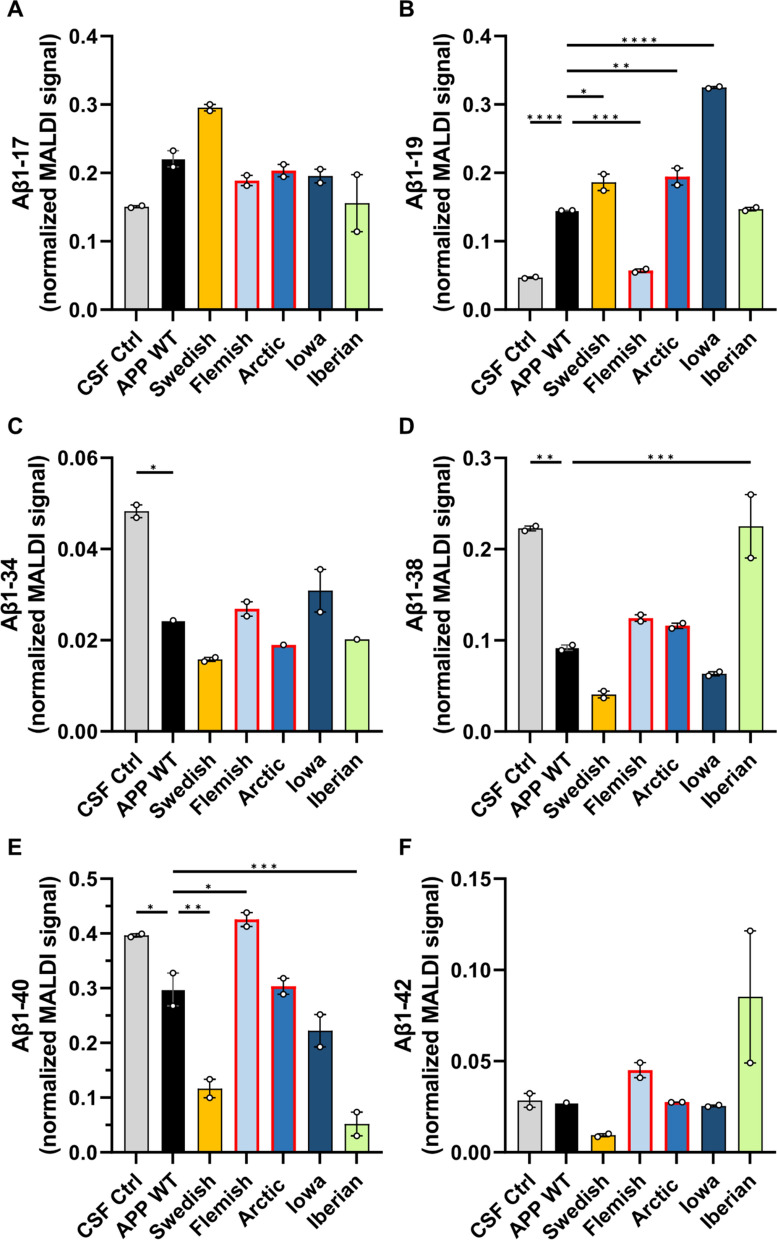
Comparison of secreted Aβ proteoforms from *APP* WT and *APP* FAD mutants. Normalized MALDI-TOF signals of **A** Aβ1-17, **B** Aβ1-19, **C** Aβ1-34, **D** Aβ1-38, **E** Aβ1-40 and **F** Aβ1-42 were plotted from the mass spectra of the *APP* WT and *APP* FAD mutant HEK293T cell media samples after IP-MALDI-TOF. The circles represent the two biological replicates (*N* = 2), each consisting of the average normalized value of two technical replicates from MALDI-TOF. Normalization was performed by dividing peak areas of each proteoforms by the sum of the peak areas of Aβ1-15, Aβ1-16, Aβ1-17, Aβ1-19, Aβ1-38, Aβ1-39 and Aβ1-40. *APP* Flemish and Arctic (marked with red bordered bars) peptides have reduced binding to 4G8 antibody. One way ANOVA with Dunnett’s post hoc test was performed for comparing each sample type to *APP* WT (*p < 0.05, **p < 0.01, ***p < 0.001, ****p < 0.0001)

*APP* Swedish showed a similar Aβ1-42/Aβ1-40 ratio (Fig. 10A) and the same peaks between Aβ 1–17 and Aβ1-38 as *APP* WT in HEK cells, but strikingly many highly elevated fragments in the first third of the Aβ spectrum, like Aβ1-14, Aβ1-15, Aβ1-16, Aβ1-17, Aβ1-19, Aβ1-20 (Figs. 8, 9), which arise most likely due to an altered secretase affinity based on the impact of the amino acid substitution KM-NL adjacent to the β-secretase cleavage site. The normalized Aβ1-17 MALDI signal was slightly greater than in *APP* WT, with also a significantly lower normalized Aβ1-40 MALDI-TOF signal (Fig. 11), also highlighted by the higher Aβ1-17/Aβ1-40 ratio (Fig. 10D).

**Fig. 12:**
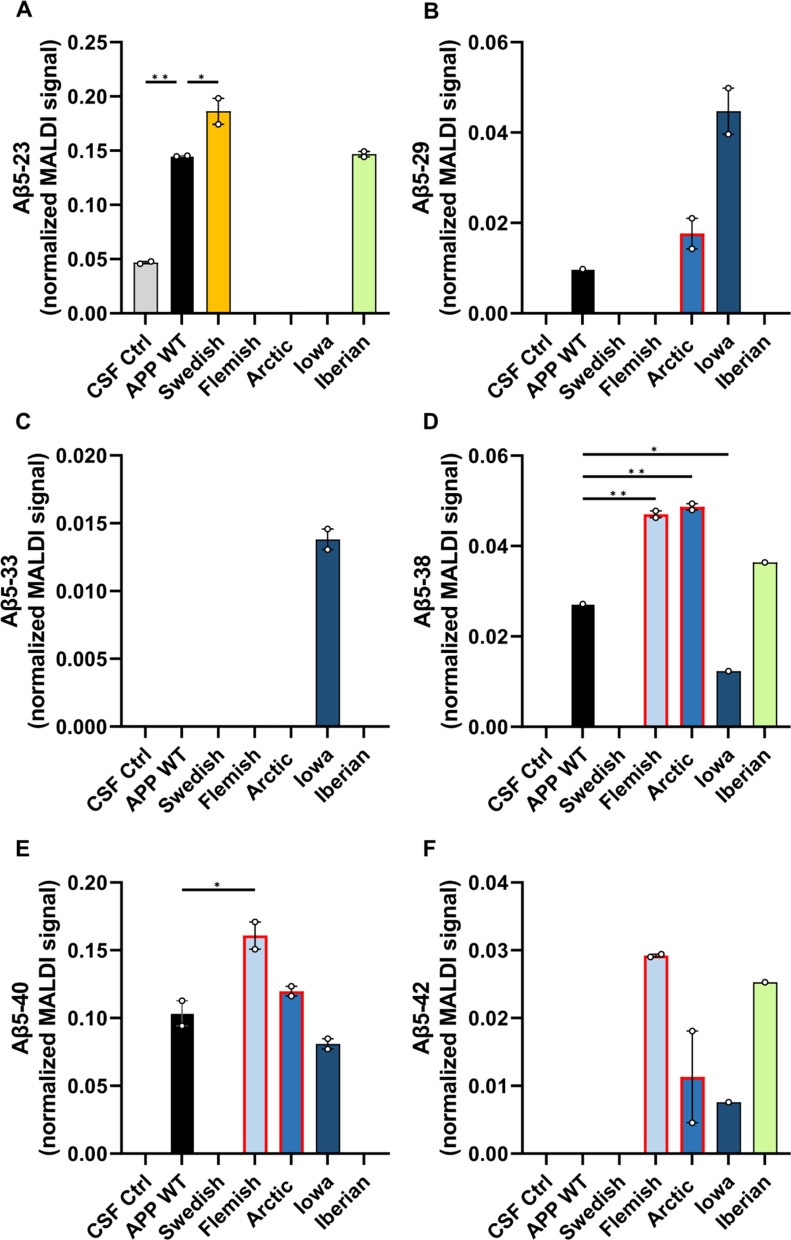
Comparison of secreted Aβ5-x from *APP* WT and *APP* FAD mutants. Normalized MALDI-TOF signals of **A** Aβ5-23 **B** Aβ5-29 **C** Aβ5-33, **D** Aβ5-38, **E** Aβ5-40 and **F** Aβ5-42 were compared between *APP* WT and *APP* FAD cell media samples from transfected HEK cells. The circles represent the two biological replicates (*N* = 2) after averaging the normalized values of two MALDI-TOF technical replicates. One way ANOVA with Dunnett’s post hoc test was performed for comparing each sample type to *APP* WT (*p < 0.05)

*APP* Iowa did not differ significantly from *APP* WT in terms of the Aβ1-42/Aβ1-40 ratio (Fig. 10A) and the normalized Aβ1-17 signal (Fig. 11A) but showed significantly higher relative Aβ1-19 signal (Fig. 11B) and a slightly lower relative Aβ1-38 signal (Fig. 11D). The *APP* Flemish profile closely resembled the *APP* WT profile in HEK cells with non-significant differences in the ratios for Aβ1-42/Aβ1-40, Aβ1-38/Aβ1-40 Aβ1-38/Aβ1-42, and Aβ1-17/Aβ1-40 (Fig. 10). Several prominent peaks between Aβ1-17 and Aβ1-38, such as Aβ1-19, Aβ1-20, Aβ1-29, and Aβ5-40, were detected along with an additional Aβ5-38 signal. The relative abundance of Aβ1-19 in the MALDI spectra was significantly lower compared to *APP* WT (Fig. 11B). The Aβ11-40 peak was found to be missing, most likely to due to the aforementioned reduced affinity of 4G8 towards *APP* Flemish and Arctic mutants. *APP* Arctic closely resembled the *APP* WT profile in HEK cells but with an additional peak detected for Aβ5-42, significantly higher normalized Aβ5-38, a complete absence of Aβ5-23 (Figs. 9, 12) and a slightly higher normalized Aβ1-19 signal (Fig. 11B). *APP* Iberian demonstrated a significant decrease in the normalized Aβ1-40 MALDI-TOF signal compared to *APP* WT (Fig. 11E) most likely explaining the significant increase in the Aβ1-42/Aβ1-40 and Aβ1-38/Aβ1-40 ratios (Fig. 10), which has been also reported earlier for this mutant [28, 29, 52]. Similarly, the Aβ1-17/Aβ1-40 ratio was also found to be higher than the other sample types (Fig. 10D). The normalized signal for Aβ1-38 was significantly greater than *APP* WT (Fig. 11D) potentially because of the lower fraction of Aβ1-40 signal in the MALDI-TOF spectrum affecting normalization. The drastic difference in the relative Aβ1-40 and Aβ1-42 signals suggests that the two proteoforms might be generated via two different product lines.

## Discussion

APP is a synaptic adhesion molecule known to mediate cell–cell adhesion via its E1 domain in the ectodomain, also between the pre- and postsynaptic side [35–37, 53]. Inhibition of ectodomain shedding via α-secretase has been shown to enhance the synaptogenic activity of APP at the presynapse, indicating that full length APP plays a major role in transsynaptic signaling. Our analyses regarding the *APP* FAD mutations and induction of presynaptic differentiation show that *APP* Iberian negatively affects presynaptic differentiation (Fig. 1). *APP* Iberian has a dramatically increased Aβ42/Aβ40 ratio and there is a wealth of data showing that this relative increase of Aβ42 enhances oligomer formation, which causes increasingly severe changes of synaptic function [54, 55]. This negative impact on synapse formation of *APP* Iberian is also reflected in our in vitro hemisynapse model, presumably via the Aβ42 oligomer formation. Hemisynapses are exposed to changes in the Aβ peptide ratio for 24 h in this assay. The short time span is most likely the reason that the other *APP* FAD mutants analyzed do not show a significant impact on presynaptic differentiation (Fig. 1), since due to conformational differences of the Aβ aggregates these forms presumably display their synaptic toxicity in more longitudinal term [49, 55, 56]. We tested via BN gel analyses if dimer or complex formation of the *APP* FAD mutants is significantly altered, but did not observe any significant differences (Fig. 2), which is in line with other studies [57]. Therefore, we assume that the transsynaptic complex of *APP* Iberian is not affected and the negative impact on presynaptic differentiation is more likely based on Aβ peptide induced synaptic toxicity.

APP is a type I transmembrane protein, which is transported in the secretory pathway whereby it can be cleaved by β-, α-, and γ-secretases [3]. Analysis of five different *APP* FAD mutations which are located at the β-secretase cleavage site (*APP* Swedish), α-secretase cleavage site (*APP* Flemish, *APP* Arctic, *APP* Iowa) or γ-secretase cleavage site (*APP* Iberian) did not reveal any significant changes compared with *APP* WT regarding their presence in the *cis*-Golgi apparatus (Fig. 4). Surprisingly, localization of *APP* Swedish was not found to be altered although this mutation had been proposed to be mainly processed in the TGN before reaching the plasma membrane [58–61]. It was demonstrated that Golgi defects can lead to increased APP processing [62]. It is even debated whether the Golgi is a site of Aβ generation [63]. The Golgi is crucial for the correct sorting and trafficking of APP and the cleaving enzymes, but our results suggest that the *APP* FAD mutations are not linked to Golgi defects. We also examined potential differences regarding the cell surface localization of the *APP* FAD mutants but did not observe any significant changes (Fig. 4).

Quantification of the localization of the *APP* FAD mutants with a marker for early endosomes, EEA1, revealed that the Iowa mutant shows a significantly higher presence in early endosomes compared with *APP* WT (Fig. 5), which was not based on a change in the internalization rate of *APP* Iowa (Fig. 6). Since endosomes are the main site for BACE1 cleavage [64, 65], a higher amount of Aβ production and a decrease in sAPPα generation for *APP* Iowa would be expected. The Iowa mutation D23N is located near the ADAM10 cleavage site, like the Arctic and Flemish mutation [19] and also lies within a cholesterol-binding site [66]. We were indeed able to show for the first time a significant decrease of sAPPα generation for *APP* Iowa (Fig. 7).

In conclusion, no significant difference regarding cellular localization has been shown for *APP* Swedish, Flemish, Arctic, Iowa and Iberian compared with *APP* WT in the *cis*-Golgi compartment and at the cell surface (Figs. 3, 4), and for *APP* Swedish, Flemish, Arctic and Iberian in early endosomes (Fig. 5).

This suggests that the differences in production of different Aβ peptides for *APP* Swedish, Flemish, Arctic and Iberian are mainly caused by the amino acid substitutions near the cleavage sites and the resulting differences in affinity of α-, β-, and γ-secretases for the different FAD mutants. *APP* FAD mutations result in varying pathological phenotypes in different mouse models, and also in patients.

### APP Swedish

AD patients carrying the APP Swedish mutation indicate atrophy with sulcal widening and mild ventricular enlargement [17]. One of the first developed AD mouse models, Tg2576, was based on the APP Swedish mutation in human APP located directly N-terminal to the β-secretase cleavage site [67]. These mice display impaired learning and memory at 9–10 month of age paralleled by increased Aβ production, formation of amyloid plaques, and a decrease in synaptic density [67, 68]. BACE1 shows a ~ 50 fold higher affinity to APP Swedish compared to APP WT [69–71] and reviewed by Armbrust et al. in 2022 [72]. We detected, as expected, a significantly increased production of the β-CTF of APP Swedish via western blot which was not accompanied by decreased α-CTF or sAPPα levels (Fig. 7). This is in line with a recent study using isogenic iPSC lines which pointed out that accumulation of the β-C-terminal fragment for APP Swedish, APP Flemish and APP V717G correlated with the size of enlarged endosomes, but not the α-C-terminal fragment [73]. They also found via an isogenic APP KO iPSC line that Rab5 + Early Endosome enlargement is dependent on APP and its processing and can be rescued by using a β-secretase inhibitor [73]. The enzyme BACE1 dominates APP Swedish processing resulting in the release of Aβ peptides starting mainly at position 1, whereas N-terminally truncated Aβ forms are only generated to a minor extent [72, 74], which is in line with data obtained by our mass spectrometric analysis of APP Swedish **(**Figs. 8, 9**)**. The mass spectrometry pattern indicated relatively higher levels of Aβ1-14, Aβ1-15, Aβ1-16, Aβ1-17, and Aβ1-19 **(**Figs. 8, 9**)** and a lower normalized Aβ1-38 signal **(**Fig. 11D**)**. In general, fragments Aβ1-37, Aβ1-38, Aβ1-39, Aβ1-40, Aβ1-42, which have been detected for APP Swedish, result from C-terminal cleavages by γ-secretase [75, 76]. Therefore, the relative decrease of Aβ1-38 reflects a modulation of γ-secretase function [77]. BACE2, the homologue of BACE1, is known to process APP in the Aβ sequence at position 19 [78]. Therefore, BACE1 and consecutive BACE2 cleavage might be responsible for generation of Aβ1-19. Fragments Aβ1-16 and Aβ1-17 most likely result from cleavages by α-secretase [76, 79]. The increase in Aβ1-17 would therefore derive from a secondary cut of the β-CTF by α-secretase. This kind of consecutive cleavages has been observed before after overexpressing β-CTF, which still yields an α-CTF in addition [39]. Furthermore, Cathepsin B has been described as an alternative protease to cut Aβ at position 1 even 440 times faster than BACE1 [80]. Additional cleavage sites of Cathepsin B within the Aβ sequence have been described at position 15/16 and 16/17 [81]. Therefore, it is tempting to speculate that fragments Aβ1-15, Aβ1-16 might be cleaved by Cathepsin B, maybe without even involving BACE1 cleavage. On the other hand, Matrix Metalloproteinase 9 (MMP9) was reported to generate different C-terminally truncated Aβ fragments, including Aβ1–16, Aβ1–20, Aβ1–23, Aβ1–30, Aβ1–33 and Aβ1–34 [82, 83].

Analysis of the different Aβ peptides is of great importance since the Aβ1-16, Aβ1-33, Aβ1-39, and Aβ1-42 expression profile in CSF enabled to differentiate patients with sporadic AD from non-demented controls with an accuracy of 86% [84]. Neprilysin has been discussed as a candidate enzyme to generate the Aβ1-33 peptide [85–87].

In general, N-terminally truncated Aβ species like Aβ2-x, Aβ3-x and Aβ4-x are derived by APP processing and are elevated in brains of AD patients with an increased aggregation propensity compared with Aβ1-x peptides [72, 88–90]. The proteases meprin β [91], cathepsin B [92] and ADAMTS4 [93] were identified as alternative β-secretases being capable of generating these N-terminally truncated Aβ species from *APP* WT, but they obviously do not play a major role in processing *APP* Swedish (Figs. 8, 9).

### APP Iberian

Increased amyloid deposition was also observed in an *APP* knock-in mouse model expressing murine APP including the humanized Aβ sequence with the Swedish and Iberian mutations compared to *APP* knock-in mice containing the Swedish mutation only [94]. The Iberian mutation significantly increases the Aβ1-42/Aβ1-40 ratio in these mice. In addition, Saito et al. showed that the mutation exacerbates loss of synapses and cognitive impairment [94].

*APP* Iberian showed no major changes regarding α- or β-secretase processing **(**Fig. 7**)**, but MALDI-TOF–MS detected higher normalized signals for Aβ1-38, Aβ1-39, and Aβ1-42, but lower normalized Aβ1-40 signal causing an increased Aβ1-42/Aβ1-40 ratio **(**Figs. 8, 9, 10, 12**)**. This is in line with results from different studies. *APP* Iberian was firstly described in an in vitro assay with a dramatically increased Aβ1-42/Aβ1-40 ratio which is mainly based on a strongly decreased Aβ1-40 production, as determined by radiolabeled immunoprecipitation followed by SDS PAGE analysis [28] and later also in different AD patients [29, 95, 96]. Besides the typical AD symptoms, *APP* Iberian was also associated with decline in motoric functions [96] and accumulation of Lewy bodies in the amygdala [95]. This could be explained by results of a recent study in mice showing that Aβ deposits can accelerate α-synuclein spreading in the brain [97] which is in line with the fact that *APP* Iberian results in a higher Aβ1-42/Aβ1-40 ratio and that Aβ1-42 is known to nucleate more efficiently than Aβ1-40 [98, 99]. Neurotoxicity of the longer Aβ fragments and cell to cell seeding could also be mediated via a non-cell autonomous mechanism such as microglial activation [100, 101]. It has been shown that reduced endogenous Aβ may also impair synaptic function [102]. Therefore, also the strongly diminished Aβ40 levels of APP Iberian could explain the negative effects of the Iberian mutation on synapse formation. In contrast, *APP* FAD mutations around the α-secretase cleavage site are reported to increase Aβ oligomerization [55, 103, 104].

### APP Flemish

Carriers of the Flemish mutation show significant amyloid accumulation in brain blood vessel walls (cerebral amyloid angiopathy; CAA) as well as parenchymal amyloid plaques [105, 106]. Thus, the clinical presentation is one involving both hemorrhagic stroke and progressive dementia [20, 25]. Though, Aβ peptides containing the *APP* Flemish mutation have relatively low aggregation propensity in vitro, including dimerization [107, 108], but show a greater abundance of Aβ1-42 paranuclei [109] and do not form structures larger than Aβ1-42 hexamers (no dodecamers) with an open and not cyclic hexamer presumably enabling penetration of blood vessel walls [56]. For *APP* Flemish, a twofold increase in Aβ1-40 [24] and Aβ1-42 production has been reported [18, 27], which is in line with our mass spectrometry results for Aβ1-40 (Figs. 8, 9, 11E). However, the difference between *APP* Flemish regarding Aβ1-42, although higher, was not significant, which might be due to the use of transfected HEK cells, where also only a mild increase of Aβ1-42 compared to other cell lines has been reported by a different study [27]. Additionally, the reduced affinity of 4G8 antibody could also be a factor. Furthermore, we were able to detect a relative decrease of the Aβ1-19 fragment (Fig. 11B) and a significant increase in Aβ5-40 (Figs. 9, 12E), which might contribute to the vascular phenotype in AD patients.

### APP arctic

For *APP* Arctic, no signs of strokes or vascular lesions were identified in AD patients, only normal AD pathology [18, 110]. Aβ1-40 peptides containing the Arctic mutation block LTP in WT mice [111] and transgenic mice expressing neuronal human APP containing the Arctic mutation show age- and dose-dependent progression of amyloid deposition in the brain and cognitive deficits regarding spatial learning and memory [112]. In vitro experiments revealed that Arctic Aβ1-40 shows increased and faster protofibril formation than WT Aβ1-40 [18] presumably based on the formation of decamers and dodecamers [56]. Enhanced protofibril formation based on the *APP* Arctic mutation has also been shown in vivo [113]. For *APP* Arctic, Aβ1-42 levels were reported to be significantly decreased while Aβ1-40 production was unchanged [18], which was not evident in the MALDI-TOF data **(**Figs. 10A, 11**)**. Aβ5-X peptides have been reported to be strongly increased in cellular models after BACE1 inhibition [114–116]; however, the presence of Aβ5-29, Aβ5-33 suggests that BACE1 is not involved in generation of these fragment.

### APP Iowa

In contrast, for AD patients carrying the Iowa mutation, cerebral amyloid angiopathy with numerous small infarcts and hemorrhages of the brain parenchyma were found [19, 117, 118]. In vitro experiments suggested that the *APP* Iowa mutation in Aβ peptides promotes fibrillogenesis of Aβ which results in greater Aβ-induced toxicity [24, 119]. For *APP* Iowa both, generation of Aβ1-40 as well as Aβ1-42 was not affected [24]. However, in the transfected HEK cell media samples, the normalized MALDI-TOF signal for Aβ1-40 was observed to be slightly lower than for the normalized *APP* WT (Fig. 11E), also resulting in a slightly higher Aβ1-42/Aβ1-40 ratio (Fig. 10A) but without much difference in the normalized Aβ1-42 signal (Fig. 11F). Additionally, N-terminally truncated Aβ peptides starting at position 5 (Aβ5-29 and Aβ5-33) were detected and the normalized Aβ1-19 signal was significantly greater than in *APP* WT (Fig. 11B) with the relative Aβ1-33 signal also appearing to be greater (Figs. 8, 9), suggesting that *APP* Iowa facilitates BACE2 and Neprilysin cleavage [76, 78].

One exciting finding of this study is that N-terminally truncated Aβ peptides starting at position 5 could be detected with a different pattern of C-terminally shortened peptides for all *APP* FAD mutants containing amino acid substitutions around the α-secretase cleavage site (*APP* Flemish, Arctic, Iowa) but not for *APP* WT, where mainly Aβ5-40 was identified, Swedish or Iberian. This suggests that these c-terminally shortened Aβ5-X peptides might contribute to the progression of AD. Indeed, it has already been shown, that Aβ5-40/42 species can be detected in some vessels with amyloid angiopathy in AD brain tissues [114]. The protease cleaving Aβ at position 5 is still unknown, but the serine protease Myelin Basic Protein (MBP) [120] and Caspases are discussed [114]. Since MBP is one of the major structural protein components of compact Myelin in the CNS [121], it might not contribute to the data of this study performed in HEK cells, though, low levels of the MBP transcript have also been identified in the kidney [122]. Aβ5-X peptides can be decreased with the α-secretase inhibitor TAPI-I and are increased in the presence of a β-secretase inhibitor [114], suggesting that a member of the ADAM protease family cleaves at position 5 of the Aβ sequence [123]. Interestingly, Aβ5-X peptides have mainly been identified to be secreted by microglia and astrocytes and not from neurons [124]

Taken together, we provide data for a detailed comparative study of five different *APP* FAD mutants regarding trafficking, proteolytic conversion including mass spectrometric data and synaptic function, which show that the amino acid substitutions of the *APP* FAD mutants have the decisive impact on their processing changes reflected in altered Aβ profiles.

Increased levels of N-terminal peptides observed in our study for most of the tested FAD mutations (Fig. 10), including Swedish, Iowa, and also Iberian. These peptides can be detected by the majority of monoclonal antibodies currently under clinical investigations, such as Aducanumab, Lecanemab and Donanemab [125, 126]. It appears reasonable that the N-terminal peptides upregulated in certain FAD mutations compete for antibody binding with longer Aβ species and Aβ protofilaments or aggregates. Thus, the different antibodies will likely exhibit higher or lower efficacy in Amyloid clearance or prevention of cytotoxicity, when tested in different mouse models and more relevant, also in clinical studies based on different FAD mutant carriers.

## Conclusion

**Fig. 13:**
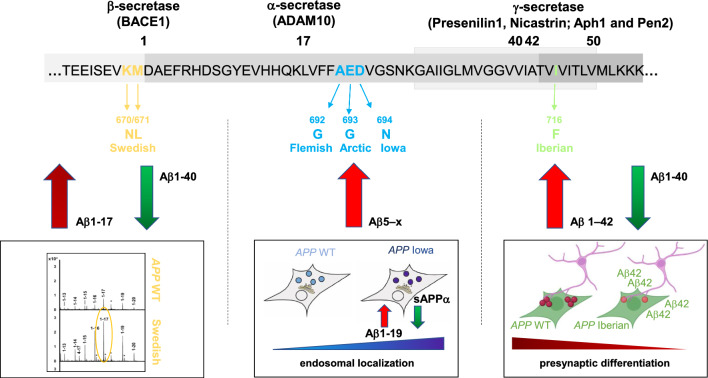
Model of different pathomechanism in the tested FAD mutants. Amino acid substitutions around the β-secretase cleavage site lead to an increase in Aβ peptides starting at position 1, mainly Aβ 1–17. The FAD mutations around the α-secretase cleavage site lead to a higher amount of N-terminally truncated Aβ peptides starting at position 5 as well as decreased sAPPα production while the mutation at the γ-secretase site showed the strongest change in the ratio of Aβ40/Aβ42. Furthermore, the APP Iberian showed a reduced synaptogenic activity whereas APP Iowa was localized to a higher amount in endosomes

Taken together, we provide data for a detailed comparative study of five different *APP* FAD mutants regarding trafficking, proteolytic conversion including mass spectrometric data and synaptic function, which show that the amino acid substitutions of the APP FAD mutants have the decisive impact on their processing changes reflected in altered Aβ profiles. We could show that the position of the different tested FAD mutants results in distinct changes in their processing. This leads to the assumption that there are also considerable differences in the underlying pathogenic mechanisms (Fig. 13). In future studies on potential therapeutic agents using mouse animal models with various sets of FAD mutations, the differences in processing of APP shown in this work should be considered.

## Abbreviations

Aβ: Amyloid beta
AD: Alzheimer’s disease
AICD: APP intracellular domain
APP: Amyloid precursor protein
APLP2: Amyloid precursor like protein 2
BACE1: β-Site amyloid precursor protein cleaving enzyme
CAA: Cerebral amyloid angiopathy
CSF: Cerebrospinal fluid
CTF: C-terminal fragments
DIV: Days in vitro
FAD: Familial Alzheimer’s disease
KO: Knockout
MBP: Myelin basic protein
PS: PSEN: Presenilin
SAM: Synaptic adhesion molecule
WT: Wild type
